# Oxidative Damage and Inflammation in Obese Diabetic Emirati Subjects

**DOI:** 10.3390/nu6114872

**Published:** 2014-11-04

**Authors:** Salah Gariballa, Melita Kosanovic, Javed Yasin, Awad El Essa

**Affiliations:** Internal Medicine, Faculty of Medicine & Health Sciences, United Arab Emirates University, Al Ain PO Box 17666, United Arab Emirates; E-Mails: melita.kosanovic@gmail.com (M.K.); javed.yasin@uaeu.ac.ae (J.Y.); a.alessa@uaeu.ac.ae (A.E.A.)

**Keywords:** obesity, visceral fat, inflammation, oxidative damage

## Abstract

Visceral obesity is more common in the Arab population and more closely related to morbidity, including diabetes and related cardiovascular diseases (CVD). Possible mechanisms that link visceral fat/obesity to diabetes and CVD complications include inflammation and increased oxidative stress; however, few data are available from the Arab population. Our aim was to determine whether increased adiposity in obese diabetic United Arab Emirates citizens is associated with sub-clinical inflammation and/or increased oxidative stress. A hundred diabetic patients who were part of a randomized controlled trial of nutritional supplements had their baseline characteristics assessed from anthropometric and clinical data following informed written consent. We used WHO figures to classify general and central obesity. Fasting blood samples were collected for the measurement of antioxidants and markers of oxidative damage and inflammation. We found that increased adiposity measured by both body mass index and waist circumference was associated with increased C-reactive protein (CRP) and decreased vitamin C after adjusting for age, duration and treatment of diabetes (*p* < 0.05). Although there is a clear trend of increased inflammatory markers, notably CRP, and decreased antioxidants with increased BMI and waist circumference in both men and women, the results are statistically significant for women only. CRP were also inversely associated with HDL. Overall, we found that BMI underestimates the rates of obesity compared to waist circumference and that increased adiposity is associated with increased inflammation and decreased HDL and antioxidant status.

## 1. Introduction

Obesity represents a major public health problem worldwide and is a major risk factor in the etiology of type 2 diabetes, hypertension and cardiovascular disease (CVD) [1,2,3]. In the Gulf region however, the prevalence of obesity is increasing rapidly and, in some of the Gulf countries, is reaching epidemic proportions [1,2,3,4]. A recent survey of United Arab Emirates (UAE) citizens revealed that the prevalence of obesity and related cardiovascular diseases is very high and, therefore, needs urgent public health attention [4]. Although increased body mass index (BMI) is used to define obesity in adults, because it correlates with the amount of their body fat and associated morbidity, recent work suggests that visceral obesity measured by waist circumference is more closely related to morbidity, especially in the Middle East and Southeast Asia [5,6,7,8,9]. Visceral fat has especially “bad” metabolic actions, because it secretes a number of inflammatory markers; some of them have been implicated in pathologies associated with obesity [3,10,11]. Possible mechanisms that relate obesity and diabetes to increased CVD risk include inflammation and oxidative damage. In obese patients, subclinical inflammation has been found to correlate with markers of oxidative stress in adipose tissue, and this may be the mechanism for obesity-related metabolic syndrome, insulin resistance and diabetes mellitus. Furthermore, both oxidative stress and low-grade inflammation may be causatively linked to the development, progression and complications of diabetes in obese patients. Although the UAE has the second highest prevalence of obesity and related diabetes mellitus in the world, at present, very little is known about the factors affecting obesity and the associated morbidities, including diabetes and CVD. In this cross-sectional analysis of the baseline data from a randomized controlled trial, we examined whether visceral fat is associated with inflammation and/or oxidative stress in obese diabetic UAE citizens.

## 2. Experimental Design

### 2.1. Methods

A hundred patients with type 2 diabetes mellitus who took part in a randomized controlled trial of antioxidants supplements were included in this analysis of baseline data. Details of the study were published elsewhere [12], but briefly, all diabetic patients regularly attending the Tawam Hospital outpatient clinics were approached and invited to take part in the study. Tawam hospital is one of the two main teaching hospitals in the city of Al Ain, serving a total population of 400,000. Inclusion criteria were patients aged 18 years and above with type 2 diabetes mellitus and who agreed to take part in the study. Individuals with severe chronic clinical or psychiatric disease, participating in other intervention trials, on dietary supplements and those unable to give informed written consent were excluded. All patients had demographic and medical data collected, including history of hypertension, smoking, alcohol and drug intake and cardiovascular diseases. The study was approved by Al Ain Medical District Human Research Ethics Committee (ethical approval number 06/62A, approved on 7 May 2007) and all subjects gave written informed consent. Anthropometric data were collected, including body weight, height, fat mass and fat-free mass, using Tanita body composition analyzer. BMI was calculated by dividing the subject’s body weight by the square of their height in meters. Waist circumference was measured using a flexible plastic tape. Whole blood samples were drawn into 2 vacutainer tubes, containing potassium EDTA as the anticoagulant. The samples were thoroughly mixed at room temperature and immediately transferred to the laboratory. Both tubes were centrifuged immediately for 10 min at 4000 rotations/min. Plasma and serum were collected and stored at −80 °C for future determination of markers of antioxidant capacity, oxidative damage and inflammation. The concentration of the lipid peroxidation product, malondialdehyde (MDA), was measured in the serum by the modified procedure of Li and Chow [13]. The content of protein-bound carbonyls, which is used to assess the extent of protein oxidation, was determined spectrophotometrically at 530 nm by the 2,4-dinitrophenylhydrazine method of Levine *et al*. [14]. Vitamins A, E and C analyses were done using HPLC, and this was performed on a Waters (Milford, MA, USA) system gradient liquid chromatography (Model 515) with an auto injector (Model 717). We used commercially available enzyme-linked immunosorbent assay (ELISA) methods to measure plasma TNF and IL6. CRP was measured by standard methods using a Synchron Clinical System (UniCel DxC-800) from Beckman Coulter, Inc. (Fullerton, CA, USA). The local Pathology Laboratory performed other routine tests, including full blood count, serum lipids, glucose, HbAic, albumin, kidney and liver function tests.

### 2.2. Statistical Analyses

Statistical analyses were performed with SPSS software, version 19.0 (SPSS Inc., Chicago, IL, USA). The nonparametric Kruskal-Wallis test was used to test between group differences, and a *p*-value < 0.05 was considered significant. Differences between groups at baseline were adjusted for age, duration and treatment of diabetes. Spearman rank correlation and the Mann-Whitney *U*-test were also used. Partial correlation was used to assess the association between inflammatory markers and lipid profile, adjusting for gender and use of statins.

## 3. Results

**Figure 1:**
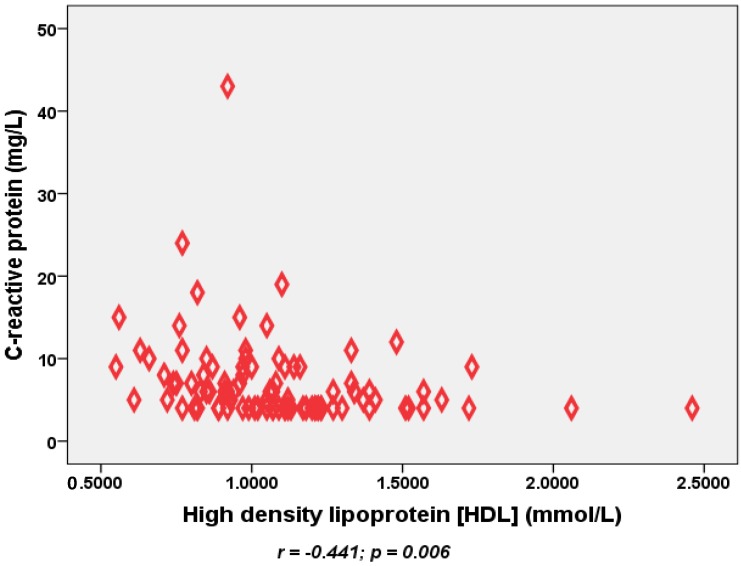
Association between the inflammatory marker C-reactive protein and high density lipoproteins in obese diabetic patients.

A total of one hundred patients with type 2 diabetes mellitus were recruited to the study. Their mean (SD) age was 51 (11) with 59 females; the mean duration of diabetes was 2.1 (0.9) years, and 74 were on statins treatment. Sixty-five patients out of the 100 were on oral hypoglycemic tables, twenty seven on insulin, five on both tablets and insulin and three controlled with diet treatment alone. Using WHO non-Asian population sex-adjusted cut-off-points for waist circumference, 72 patients out of 100 would be at high health risk and 20 patients at increased health risk. Corresponding numbers for BMI cut-off-points were 60 (BMI ≥ 30) and 30 (BMI ≥ 25), respectively. Table 1 shows the baseline characteristic and CVD risk factor distribution according to the BMI cut-off-points for determining health actions. Waist and hip circumferences were significantly higher and HDL lower in people at increased and high health risks compared with those with satisfactory health risks. Patients with high health risks (BMI ≥ 30) were also non-significantly younger compared with those with satisfactory health risks (Table 1). Table 2, Table 3, Table 4 and Table 5 show inflammation, antioxidant and oxidative damage markers according to BMI and waist circumference cut-off-points for determining health actions for both men and women. There is clear trend of increased inflammatory markers and decreased antioxidants, notably for CRP with increased BMI and waist circumference in both men and women; however, the results are statistically significant for women only. For example, CRP is significantly higher and vitamin C levels lower in women with increased adiposity (*p* > 0.05) (Table 3 and Table 5). We found no significant associations between markers of oxidative damage and adiposity. CRP also showed an inverse correlation with HDL (*r* = −0.44, *p* = 0.006) (Figure 1). The association between CRP and HDL remains significant after adjusting for gender (*r* = −0.27, *p* = 0.009) and use of statins (*r* = 0.24, *p* < 0.020). Associations between other inflammatory and lipid profile markers were not statistically significant.

**Table 1:** Baseline characteristics according to the BMI cut-off-points for non-Asian populations (mean SE).

Variable	Body Mass Index (BMI) ^†^		
	Satisfactory (*n =* 10)	Increased Risk (*n =* 30)	High Risk (*n =* 60)
Age (year)	56 (4)	54 (2)	49 (1)
Male:female (*n*)	7:3	15:15	19:41
Smoking (*n*)	2	1	5
Duration of diabetes (year)	1.9 (0.4)	2.3 (0.2)	2 (0.1)
Previous ischemic heart disease (*n*)	0	5	9
Previous cerebrovascular disease (*n*)	0	1	0
Previous hypertension (*n*)	6	18	38
Total number of drugs/patient	2.2	2.1	2.0
Number of patients taking statins	9	22	43
Systolic BP (mmHg)	142 (6)	138 (4)	133 (2)
Diastolic BP (mmHg)	79 (5)	78 (2)	80 (1)
High cholesterol	7	25	42
Waist circumference * (cm)	91 (3.5)	95 (1.4)	110 (1.5)
Hip circumference * (cm)	95 (3.1)	101 (1.2)	118 (1.3)
HbA1c (%)	7.97 (0.8)	7.95 (0.5)	8.1 (0.2)
Total cholesterol (mmol/L)	4.55 (0.3)	4.46 (0.2)	4.55 (0.1)
LDL (mmol/L)	2.8 (0.29)	2.8 (0.19)	2.9 (0.12)
HDL * (mmol/L)	1.24 (0.1)	1.04 (0.1)	1.07 (0.04)
Triglycerides (mmol/L)	1.0 (0.14)	1.39 (0.16)	1.46 (0.15)

^†^ BMI categories: satisfactory: 18.5–24.9; increased risk: 25–29.9; high risk: ≥30. * *p*-Value ≤ 0.05.

**Table 2:** Antioxidants and markers of oxidative damage and inflammation according to the WHO BMI cut-off-points for male subjects (mean SE).

Variable	BMI			
	Satisfactory (*n =* 7)	Increased Risk (*n =* 15)	High Risk (*n =* 19)	*p*-Value
CRP (mg/L) ^†^	4.6 (0.4)	5.8 (0.6)	6.5 (0.7)	0.250
IL6 (pg/mL)	3.03 (0.42)	3.87 (0.42)	3.33 (0.37)	0.476
TNFα (pg/mL)	1.13 (0.25)	1.63 (0.40)	3.45 (1.14)	0.667
Vitamin C (mg/L)	23.3 (12)	27.3 (5)	20.6 (3)	0.382
Vitamin E (mg/L)	9.5 (2.2)	10.4 (1.0)	8.1 (1.2)	0.233
Vitamin A (mg/L)	0.98 (0.28)	0.88 (0.1)	0.75 (0.1)	0.577
Protein carbonyl (nmol/mg)	0.47 (0.18)	0.71 (0.1)	0.48 (0.1)	0.192
MDA (nM/mL) ^†^	11.8 (8.1)	11.6 (2.8)	5.45 (1.2)	0.474

^†^ CRP, C-reactive proteins; MDA, malondialdehyde.

**Table 3:** Antioxidants and markers of oxidative damage and inflammation according to the WHO BMI cut-off-points for female subjects.

Variable	BMI			
	Satisfactory (*n =* 3)	Increased Risk (*n =* 15)	High Risk (*n =* 41)	*p*-Value
CRP ^†^	4.3 (0.3)	5.9 (0.9)	10.0 (1.3)	0.020 *
IL6	3.15 (0.52)	3.11 (0.46)	2.93 (0.51)	0.773
TNFα	1.19 (0.9)	1.32 (1.8)	2.04 (3.3)	0.767
Vitamin C	55.8 (3.6)	30.7 (4.7)	29.6 (2.8)	0.080
Vitamin E	9.4 (3.8)	5.7 (0.8)	8.5 (0.6)	0.031 *
Vitamin A	0.73 (0.25)	0.46 (0.09)	0.61 (0.05)	0.082
Protein carbonyl	0.94 (0.06)	0.76 (0.07)	0.65 (0.07)	0.334
MDA ^†^	8.9 (1.8)	17.3 (4.5)	11.8 (1.9)	0.270

^†^ CRP, C-reactive proteins; MDA, malondialdehyde. * *p*-Value ≤ 0.05.

**Table 4:** Antioxidants and markers of oxidative damage and inflammation according to the WHO waist circumference cut-off-points for male subjects (mean SE).

Male	Waist Circumference			
	Satisfactory (*n =* 5)	Increased Risk (*n =* 15)	High Risk (*n =* 21)	*p*-Value
CRP	4.8 (0.6)	4.8 (0.3)	7.0 (0.7)	0.065
IL6	3.39 (0.5)	3.36 (0.4)	3.58 (0.4)	0.874
TNFα	0.87 (0.3)	3.39 (1.4)	2.04 (0.5)	0.338
Vitamin C	33.8 (14)	23 (0.5)	21 (4)	0.781
Vitamin E	8.7 (2)	10.5 (1)	8.3 (1)	0.225
Vitamin A	1.1 (0.4)	0.93 (0.07)	0.70 (0.1)	0.157
Protein carbonyl	0.598 (0.16)	0.527 (0.12)	0.582 (0.09)	0.967
MDA	17.2 (11)	7.7 (2.7)	7.6 (1.6)	0.787

Waist circumference categories: satisfactory: ≤92 cm; increased risk: 93–102 cm; high risk: >102 cm.

**Table 5:** Antioxidants and markers of oxidative damage and inflammation according to the WHO waist circumference cut-off-points for female subject (mean SE).

Female	Waist Circumference						
	Satisfactory (*n =* 3)		Increased Risk (*n =* 5)		High Risk (*n =* 51)		*p*-Value
CRP	4 (0.0)		4.6 (0.4)		9.4 (1)		0.021 *
IL6	2.85 (0.4)		4.55 (1.0)		2.84 (0.3)		0.131
TNFα	0.76 (0.4)		1.52 (0.5)		1.90 (0.4)		0.714
Vitamin C	54.7 (5)		45.6 (9)		28.4 (3)		0.016 *
Vitamin E	6.2 (3)		9.3 (2)		7.8 (0.5)		0.563
Vitamin A	0.4 (0.1)		0.57 (0.2)		0.59 (0.1)		0.734
Protein carbonyl	0.870 (0.13)		0.890 (0.1)		0.664 (0.06)		0.247
MDA	7.56 (0.9)		12.2 (2.6)		13.03 (2)		0.719

Waist circumference categories: satisfactory: ≤80 cm; increased risk: 81–88 cm; high risk: >88 cm; * *p*-value ≤ 0.05.

## 4. Discussion

Although BMI is used to define obesity in adults worldwide, we found that using waist circumference cut-off-points, as suggested by WHO, for non-Asian populations, the proportion classified as obese increased significantly in our patients compared to using the BMI cut-off-points [7]. Similar results were obtained when we used the waist circumference cut-off-points reported for Iranian adults by Esteghamati *et al*. [15]. This is an important finding, given the results of the recent INTERHEART study, in which the relation between BMI and waist circumference to myocardial infarction was assessed in 27,098 participants from 52 countries, reporting that the waist-to-hip ratio showed the strongest relationship with myocardial infarction worldwide [8,9]. Furthermore, if a raised waist/hip ratio were to be used to assess the risk of cardiovascular disease, as suggested by the INTERHEART data, the proportion classified as obese worldwide would increase substantially, especially in the Middle East and Southeast Asia [8]. The INTERHEART data went further by suggesting that the BMI seems to be of no value in several populations, such as Arabs or people from southern Asia [8,9]. We also found significant positive correlations between adipose tissue measured by either the BMI or waist circumference and markers of inflammation independent of diabetic control status or medications. Furthermore, low-grade inflammation is negatively correlated with HDL and markers of antioxidant status. The role of low-grade inflammation in the onset and progression of chronic diseases, including obesity, type 2 diabetes and CVD, is receiving increasing attention. Both oxidative stress and low-grade inflammation may be causatively linked to the development, progression and complications of diabetes in obese patients. Recent research does indeed support a close link between oxidative stress and diabetes evolution, revealing that oxidative stress occurs before the appearance of clinical manifestations of late diabetic complications, suggesting a key role in the pathogenesis of the disease [16]. In addition, a number of studies have reported an association between oxidative stress and insulin resistance and that some antioxidants may improve insulin resistance [17,18]. Factors that reduce oxidative stress and attenuate inflammation could provide an important tool to reduce the burden associated with obesity and related chronic disease, including diabetes and CVD. Dietary supplements with antioxidant and anti-inflammatory effects may present a novel strategy of controlling and reducing the complications of obesity at the population level. A recent systematic review and meta-analysis has reported that increasing daily intake of green leafy vegetables could significantly reduce the risk of type 2 diabetes, and this should be investigated further [19]. Another recent data also suggest that dietary antioxidant intake may be a predictor of the risk of developing metabolic syndrome features, such as adiposity or impairments in systolic blood pressure, serum glucose and free fatty acids, as well as some inflammatory biomarkers in healthy subjects [20]. The low HDL levels in people with increased adiposity and the significant inverse association with increased inflammation is also interesting given the already known association between low levels of HDL and increased risk of CVD and also the reduced protective effects of HDL in patients with type 2 diabetes [21]. The lack of association between inflammation and other lipid profile markers is most likely explained by the high number of patients already taking statins and that the currently used statins do not affect the levels of HDL very much compared to their main action of reducing LDL-cholesterol.

Although our study sample is small, we are not, however, aware of similar data from the area. Nevertheless, the UAE society and other similar nations in the Middle East have been through rapid socioeconomic and social changes with urbanization over the last 40 years. Accompanying changes in diet and lifestyle are therefore leading to a growing epidemic of being overweight/obesity, type 2 diabetes and other related CVDs. Studies of the early mechanisms of oxidative damage and subclinical inflammation that link obesity and its complications, including diabetes and CVD, and the benefits of antioxidants found in fruit and vegetables known to promote health by combating oxidative stress and inflammation are urgently needed in this population to help reduce the burden of these diseases, which have already reached epidemic proportions.

## 5. Conclusions

In conclusion, we found that BMI underestimates the rates of obesity compared to waist circumference and that increased adiposity is associated with increased inflammation and decreased HDL and antioxidant status. A larger clinical trial is needed to find out whether higher intakes of antioxidants found in fruit and vegetables in this population would help to reduce the burden of obesity and its complications, including diabetes and CVD.

## References

[1] World Health Orgnization (2000). Obesity: Preventing and managing the global epidemic. Report of a WHO consultation. World Health Org. Tech. Rep. Ser..

[2] McLellan F. (2002). Obesity rising to alarming levels around the world. Lancet.

[3] British Nutrition Foundation (2005). The report of a British nutrition foundation task force. Cardiovascular Disease, Diet, Nutrition and Emerging Risk Factors.

[4] Malik A., Babir A., Abi Saab B., Roglic G., King H. (2005). Glucose intolerance and associated factors in the UAE. Diabetes Res. Clin. Pract..

[5] Baik I., Ascherio A., Rimm E.B., Giovannucci E., Spiegelman D., Stampfer M.J., Willett W.C. (2000). Adiposity and mortality in men. Am. J. Epidemiol..

[6] Rexode K.M., Carey V.J., Hennekens C.H., Walters E.E., Colditz G.A., Stampfer M.J., Willett W.C., Manson J.E. (1998). Abdominal adiposity and coronary heart disease in women. JAMA.

[7] WHO Expert Consultation (2004). Appropriate body-mass index for Asian populations and its implications for policy and intervention strategies. Lancet.

[8] Yusuf S., Hawken S., Ounpuu S., Baustista L., Franzosi M.G., Commerford P., Lang C.C., Rumboldt Z., Onen C.L., Lisheng L. (2006). Obesity and risk of myocardial infarction in 27,000 participants from 52 countrries: A case-control study. Lancet.

[9] Yusuf S., Hawken S., Ounpuu S., Dans T., Avezum A., Lanas F., McQueen M., Budaj A., Pais P., Varigos J. (2004). Effect of potentially modifiable risk factors associated with myocardial infarction in 52 countries (the INTERHEART study): Case-control study. Lancet.

[10] Trayhurn P. (2005). The bology of obesity. Proc. Nutr. Soc..

[11] Pouliot M.C., Despres J.P., Lemieux S., Moorjani S., Bouchard C., Tremblay A., Nadeau A., Lupien P.J. (1994). Waist circumference and abdominal sagittal diameter: Best simple anthropometric indexes of abdominal visceral adipose tissue accumulation and related cardiovascular risk in men and women. Am. J. Cardiol..

[12] Gariballa S., Afandi B., Abuhaltem M., Yassin J., Habib H., Ibrahim W. (2013). Oxidative damage and inflammation in obese diabetic Emirati subjects supplemented with antioxidants and B-vitamins. Nutr. Metab. (Lond.).

[13] Li X.Y., Chow C.K. (2004). An improved method for the measurement of malondialdehyde in biological samples. Lipids.

[14] Levine R.L., Garland D., Oliver C.N., Amici A., Climent I., Lenz A., Ahn B., Shaltiel S., Stadtman E. (1990). Determination of carbonyl content in oxidatively modified proteins. Methods Enzymol..

[15] Esteghamati A., Ashraf H., Rashidi A., Meysamie A. (2008). Waist circumference cut-off points for the diagnosis of metabolic syndrome in Iranian adults. Diabetes Res. Clin. Pract..

[16] Hill M.F. (2008). Emerging role for antioxidant therapy in protection against diabetic cardiac complications: Experimental and clinical evidence for utilization of classic and new antioxidants. Curr. Cardiol. Rev..

[17] Evans J.L. (2007). Antoxidants: Do they have a role in the treatment of insulin resistance?. Indian J. Med. Res..

[18] Evans J., Goldfine I.D., Maddux B.A., Grodsky G.M. (2002). Oxidative stress and stress activated signalling pathways: A unifying hypothesis of type 2 diabetes. Endocr. Rev..

[19] Carter P., Gray L.J., Troughton J., Khunti K., Davies M.J. (2010). Fruit and vegetable intake and incidence of type 2 diabetes mellitus: Systematic review and meta-analysis. BMJ.

[20] Puchau B., Zulet A., Gonzalez A., Hermsdorff H., Martinez J. (2010). Dietary total antioxidant capacity is negatively associated with some metabolic syndrome features in healthy young men. Nutrition.

[21] Barter P. (2013). High density lipoprotein: A therapeutic target in type 2 diabetes. Endocrinol. Metab..

